# Drugst.One - A plug-and-play solution for online systems medicine and network-based drug repurposing

**Published:** 2023-07-04

**Authors:** Andreas Maier, Michael Hartung, Mark Abovsky, Klaudia Adamowicz, Gary D. Bader, Sylvie Baier, David B. Blumenthal, Jing Chen, Maria L. Elkjaer, Carlos Garcia-Hernandez, Mohamed Helmy, Markus Hoffmann, Igor Jurisica, Max Kotlyar, Olga Lazareva, Hagai Levi, Markus List, Sebastian Lobentanzer, Joseph Loscalzo, Noel Malod-Dognin, Quirin Manz, Julian Matschinske, Miles Mee, Mhaned Oubounyt, Alexander R. Pico, Rudolf T. Pillich, Julian M. Poschenrieder, Dexter Pratt, Nataša Pržulj, Sepideh Sadegh, Julio Saez-Rodriguez, Suryadipto Sarkar, Gideon Shaked, Ron Shamir, Nico Trummer, Ugur Turhan, Ruisheng Wang, Olga Zolotareva, Jan Baumbach

**Affiliations:** 1 Institute for Computational Systems Biology, University of Hamburg, Hamburg, Germany; 2 Division of Orthopaedic Surgery, Schroeder Arthritis Institute, and Data Science Discovery Centre, Osteoarthritis Research Program, Krembil Research Institute, UHN, Toronto, Canada; 3 Data Science Discovery Centre for Chronic Diseases, Krembil Research Institute, University Health Network, 60 Leonard Avenue, 5KD-407, Toronto, ON, M5T 0S8, Canada; 4 Department of Molecular Genetics, University of Toronto, Toronto, ON, Canada; 5 The Donnelly Centre, University of Toronto, Toronto, ON, Canada; 6 Department of Computer Science, University of Toronto, Toronto, ON, Canada; 7 Princess Margaret Cancer Centre, University Health Network, Toronto, ON, Canada; 8 The Lunenfeld-Tanenbaum Research Institute, Mount Sinai Hospital, Toronto, ON, Canada; 9 Chair of Experimental Bioinformatics, TUM School of Life Sciences, Technical University of Munich, Munich, Germany; 10 Department Artificial Intelligence in Biomedical Engineering (AIBE), Friedrich-Alexander University Erlangen-Nürnberg (FAU), 91052 Erlangen, Germany; 11 Department of Medicine, University of California San Diego, 9500 Gilman Drive, La Jolla, CA, 92093, USA; 12 Department of Neurology, Odense University Hospital, Odense, Denmark; 13 Institute of Clinical Research, University of Southern Denmark, Odense, Denmark; 14 Institute of Molecular Medicine, University of Southern Denmark, Odense, Denmark; 15 Barcelona Supercomputing Center (BSC), 08034 Barcelona, Spain; 16 Institute for Advanced Study (Lichtenbergstrasse 2a, D-85748 Garching, Germany), Technical University of Munich, Germany; 17 National Institute of Diabetes, Digestive, and Kidney Diseases, Bethesda, MD 20892, United States of America; 18 Departments of Medical Biophysics and Computer Science, University of Toronto, Toronto, Canada; 19 Institute of Neuroimmunology, Slovak Academy of Sciences, Bratislava, Slovakia; 20 Division of Computational Genomics and Systems Genetics, German Cancer Research Center (DKFZ), 69120 Heidelberg, Germany; 21 Junior Clinical Cooperation Unit Multiparametric methods for early detection of prostate cancer, German Cancer Research Center (DKFZ), Heidelberg, Germany; 22 European Molecular Biology Laboratory, Genome Biology Unit, 69117 Heidelberg, Germany; 23 Blavatnik School of Computer Science, Tel-Aviv University, Tel-Aviv, Israel; 24 Heidelberg University, Faculty of Medicine, and Heidelberg University Hospital, Institute for Computational Biomedicine, Bioquant, Heidelberg, Germany; 25 Department of Medicine, Brigham and Women’s Hospital, Harvard Medical School, Boston, MA 02115, USA; 26 Institute of Data Science and Biotechnology, Gladstone Institutes, 1650 Owens Street, San Francisco, 94158, California, USA; 27 Department of Computer Science, University College London, London WC1E 6BT, UK; 28 ICREA, Pg. Lluís Companys 23, 08010 Barcelona, Spain; 29 Department of Clinical Genetics, Odense University Hospital, Odense, Denmark; 30 Computational Biomedicine Lab, Department of Mathematics and Computer Science, University of Southern Denmark, Odense, Denmark

**Keywords:** Drug repurposing, Systems medicine, Interactive network enrichment, Biomedical network exploration, Network integration, Biomedical data analysis, Data visualization

## Abstract

In recent decades, the development of new drugs has become increasingly expensive and inefficient, and the molecular mechanisms of most pharmaceuticals remain poorly understood. In response, computational systems and network medicine tools have emerged to identify potential drug repurposing candidates. However, these tools often require complex installation and lack intuitive visual network mining capabilities. To tackle these challenges, we introduce Drugst.One, a platform that assists specialized computational medicine tools in becoming user-friendly, web-based utilities for drug repurposing. With just three lines of code, Drugst.One turns any systems biology software into an interactive web tool for modeling and analyzing complex protein-drug-disease networks. Demonstrating its broad adaptability, Drugst.One has been successfully integrated with 21 computational systems medicine tools. Available at https://drugst.one, Drugst.One has significant potential for streamlining the drug discovery process, allowing researchers to focus on essential aspects of pharmaceutical treatment research.

## Introduction

In recent years, rapid technological advancements and unmet medical needs have fueled the development of computational tools that leverage systems biology methodologies to decipher complex biomedical data [1]. These tools frequently target the identification of specific proteins or genes in a given disease context, such as marker genes indicative of disease progression [2,3]. The visualization of these results in a biomedical network context can greatly improve their interpretability, allowing us to better understand the underlying disease mechanisms and the interrelationships among the identified entities [4]. This principle applies to a variety of biomedical fields, including oncology [5,6], virology [7], and disease subtype identification and patient stratification through differential gene expression analysis [8,9]. Rendering these intricate cellular processes as graphs aids researchers in tailoring more precise pharmaceutical treatments, minimizing side effects [10,11] and opening prospects for novel therapeutic and diagnostic strategies, such as mechanistic drug repurposing [12].

Key challenges in the development of systems biology platforms include the integration of comprehensive biomedical data and the creation of flexible graphical user interfaces for data analysis, prioritization, and visualization. Stand-alone software such as Cytoscape [13] visualizes biological networks but necessitates local installation for each user. To circumvent this, developers often provide online solutions dependent solely on browser compatibility. However, this presents additional hurdles for researchers who may lack sufficient web-development skills and need to establish and maintain an infrastructure, including a server hosting a database and a website. Beyond network visualization, the collection, harmonization, integration, and incorporation of diverse biomedical data demand a significant time investment [14]. Moreover, the database should be maintained and regularly updated, a chore that is often not addressed by bioinformatics tools that primarily provide a result overview with a limited set of features. Thus, if network exploration is not neglected due to the additional workload, unique solutions are being developed from scratch, resulting in network visualizers and explorers of varying quality [7,8,15].

**Figure 1:**
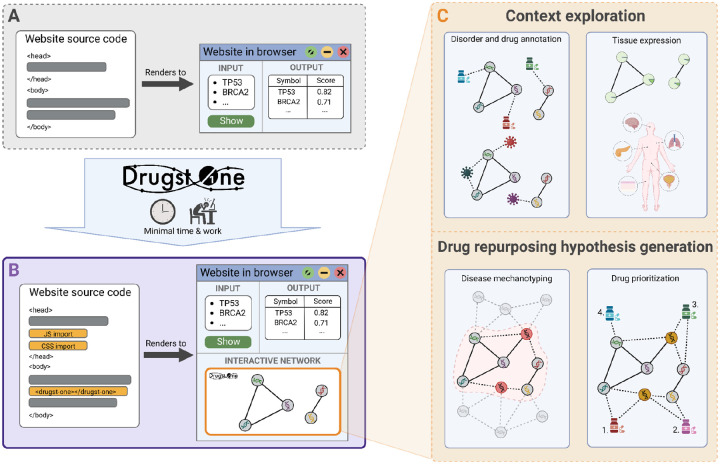
Drugst.One enables web developers to add a fully functional network explorer to any website with minimal coding effort (biomedical web tool before (**A**) and after (**B**) Drugst.One integration). (**C**): A network can be explored manually or by using network medicine algorithms to identify disease mechanisms and drug repurposing candidates. Associated diseases and tissue-specific expression are additional information layers to gain insight into the network context.

We developed Drugst.One to reduce software engineering overhead, bundle development capacities, and to standardize and simplify network analysis and visual network exploration for biomedical web tools (Figure 1). With minimal programming effort, Drugst.One can turn any gene or protein-based systems biology tool into a powerful online toolkit for network integration and visualization, as well as mechanistic drug repurposing. Drugst.One is a customizable plug-and-play solution for web-application developers in need of a feature-rich network explorer coupled with a biomedical protein-drug-disease network data warehouse. With as little as three lines of code, Drugst.One can be added to any biomedical web tool, highlighting opportunities for drug repurposing and elucidating disease mechanisms. Incorporating multiple state-of-the-art databases (see Supplementary Table 1) to complement visualized data, Drugst.One provides an intuitive interface for applying algorithms for exploratory network analyses, drug target and drug repurposing candidate identification and prioritization. Weekly updates guarantee the relevance of its database for frequently changing data. Currently, Drugst.One is integrated into 21 systems medicine software resources (Table 1), including mirDIP (see Supplementary Figure S3) [16] and WikiPathways [17]. In this article, we describe the functionality of Drugst.One and demonstrate its utility on the basis of two studies – on drug repurposing for inflammatory bowel disease (IBD) and on exploring the smooth muscle cell (SMC) proliferation pathway.

## Results

### Drugst.One overview

Drugst.One closes the gap between disease mechanism mining and hypothesis generation for drug repurposing. The required input is a list of proteins or genes in HGNC, UniProt, Ensembl, or Entrez ID space. On demand, these entities are integrated into the interactome and automatically annotated with clinically relevant information, e.g., targeting drugs or known disease associations. Exploratory functions allow the visualization of known drug indications and disease associations as well as an overlay for tissue-specific expression information (Figure 1C). For most information-enriching functions, Drugst.One provides several data sources to choose from (Supplementary Notes 3.1). Convenience features for network control (such as enabling the interactive mode or resetting the view) and export are available to assist exploratory analysis further.

Drugst.One originates from the network-based drug repurposing platforms CoVex [7] and CADDIE [5], developed for the application in SARS-CoV-2 and cancer, respectively. While they provide disease-specific information, both tools share underlying principles and algorithms. These tested and published methods form the Drugst.One algorithmic toolkit for more extensive analysis. Module identification algorithms provide means to identify additional potential drug targets from the interactome to enrich the mechanistic context. In a second step, drugs that are directly or indirectly linked can be ranked. This allows the assessment of the compound’s potential to be repurposed using network-based algorithms. Although both steps work automatically, users can infuse their expert knowledge by adjusting input gene sets. Users can choose among seven drug prioritization and drug target identification algorithms to rank small molecules directly or indirectly targeting disease proteins, thus serving as potential drug repurposing candidates (Supplementary Notes 3.2).

Overrepresentation analysis using g:Profiler [18] or functional coherence validation using DIGEST [19] on all loaded proteins in the network can be run with one click. Further, searching for curated pathways containing the same proteins in NDEx IQuery [20] allows for even more interoperability. A full list of projects partnering with the ‘Drugst.One Initiative’ by integrating Drugst.One can be found in Table 1. Collaborators that assisted and provided technologies that helped to build features in Drugst.One can be found in Supplementary Notes 4.

### Drugst.One integration and customization

**Figure 2:**
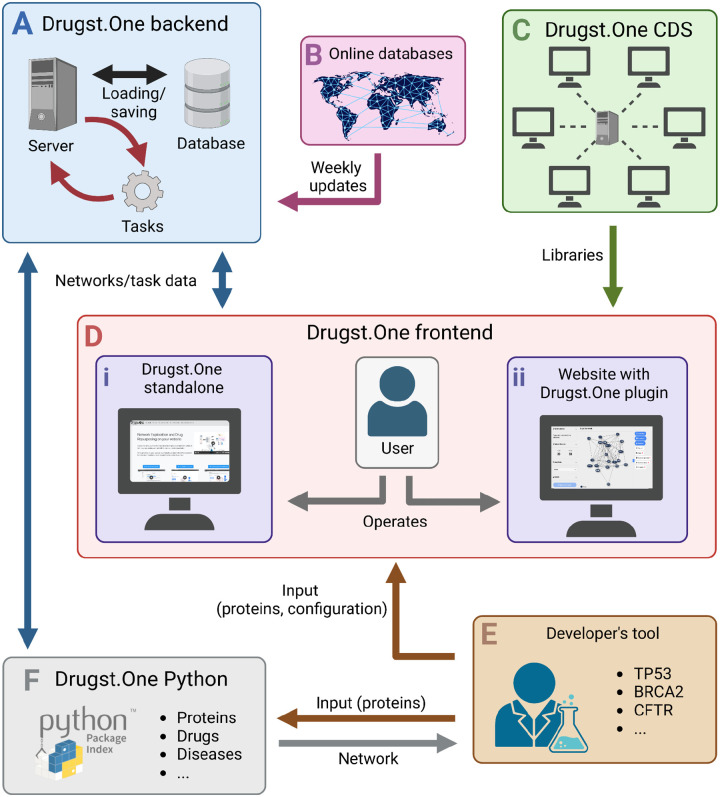
The Drugst.One ecosystem: The Drugst.One server (**A**) updates weekly from online databases (**B**), executes computationally demanding tasks, and provides data to the Drugst.One plugin (**D i** and **D ii**). The frontend is loaded from the content delivery system (CDS), (**C**), receives the network data from the developer integrating Drugst.One (**E**), and presents it to the user. Drugst.One can also be accessed programmatically through a Python package (**F**).

The Drugst.One ecosystem is a multi-component platform consisting of a website, the web plugin, a server, a content delivery system (CDS), and a Python package, as depicted in Figure 2 (for details, see Supplementary Notes 1).

The web plugin can be added to any webpage by importing one JavaScript and one stylesheet file from the https://cdn.drugst.one distribution server, and by adding the ‘drugst-one’ HTML tag to the source code of any system medicine tool’s website (Supplementary Figure S2). Features can be customized to a high degree through JSON configuration strings that are passed as attributes. This includes default states of on/off toggles, the network, and the node and edge groups that define the network style. The plugin is responsive to changes during runtime, allowing developers to add buttons or other controls to the host page, for example switching between networks. For seamless integration of the rendered plugin into any website, styling and coloring are controllable by adding specific CSS variables to the website stylesheet. To assist developers in the integration process, the Drugst.One website provides conclusive documentation of available parameters, features, and styles. It further offers an interactive configuration page at https://drugst.one/playground where configuration options are categorized, and the replication of a configured Drugst.One instance is achieved by simple copy-pasting of the generated code snippets to the developers’ websites. This low-code approach allows bioinformatics researchers to provide the community with an interactive mechanism mining web tool within hours or even minutes instead of days. The lightweight Drugst.One JavaScript library connects to the Drugst.One data warehouse server, which handles all the computationally expensive work like data annotation, mapping, and asynchronous algorithm execution.

Alternatively, a standalone integration of Drugst.One is provided at https://drugst.one/standalone, which can be accessed and customized using URLs or POST-based requests. This way, results from any website or even a command line tool can be redirected to Drugst.One through a simple web service request (Supplementary Notes 2). Detailed documentation about all Drugst.One integration options can be found at https://drugst.one/doc.

### Drugst.One integration examples

#### Drugst.One plugin integration with ROBUST-Web

ROBUST-Web (https://robust-web.net) presents a modified version of ROBUST [31] in an online web interface. It provides a network-based disease module identification algorithm based on prize-collecting Steiner trees that mitigates study bias using edge costs derived from study-attention or bait-usage information. Given a set of seed genes and a PPI network, ROBUST-Web constructs disease modules and passes nodes and edges to the Drugst.One plugin that takes care of result presentation and visualization in an interactive network view. Drugst.One also serves as a network explorer for the analysis of modules by offering an estimation of functional coherence with DIGEST [19], GO enrichment with g:Profiler, or a lookup in NDEx IQuery for identifying pathways with the same participants. Additionally, it adds disease annotations and drug repurposing functions to make the results of ROBUST-Web more actionable and derive hypotheses for follow-up research.

#### Drugst.One plugin integration with BiCoN

BiCoN [8] is a systems medicine tool for simultaneous patient stratification and disease mechanism identification, i.e., network-based endotyping. BiCoN uses a molecular interaction network as input and identifies two subgroups of patients along with a subnetwork that is enriched for differentially expressed genes between the two groups. These subnetworks can serve as composite biomarkers but may also be enriched for putative drug targets. Since BiCoN also features a web version (https://exbio.wzw.tum.de/bicon), we integrated the Drugst.One plugin for enhancing the result presentation by interactively visualizing the identified subnetworks. This allows users to explore possible drug repurposing candidates targeting the newly identified disease mechanisms, which can subsequently be experimentally validated.

#### Drugst.One link-out from WikiPathways

WikiPathways [32] is a widely used, community-driven platform for exploring molecular pathways. It allows users to upload, edit, browse, and download a constantly growing pool of pathway datasets. Pathway data can be used to identify and understand key players in metabolism, which is critical for understanding rare or common diseases such as COVID-19 [32]. Thus, pathways allow for the prediction of drug target and drug repurposing candidates and are commonly used in the development of new disease treatments [33]. When inspecting individual pathways on the WikiPathways platform, users now have the option to forward the pathway genes to the Drugst.One standalone version by clicking a ‘Query Drugst.One’ link now provided by WikiPathways, located in the search menu of the ‘Participants’ table. The link redirects the user to the Drugst.One website, visualizing pathway genes, drugs directly targeting them, and offering the complete toolset of Drugst.One. In the following, we give an example of the Drugst.One usage for exploration of the smooth muscle differentiation and proliferation pathway (WP1991).

**Figure 3:**
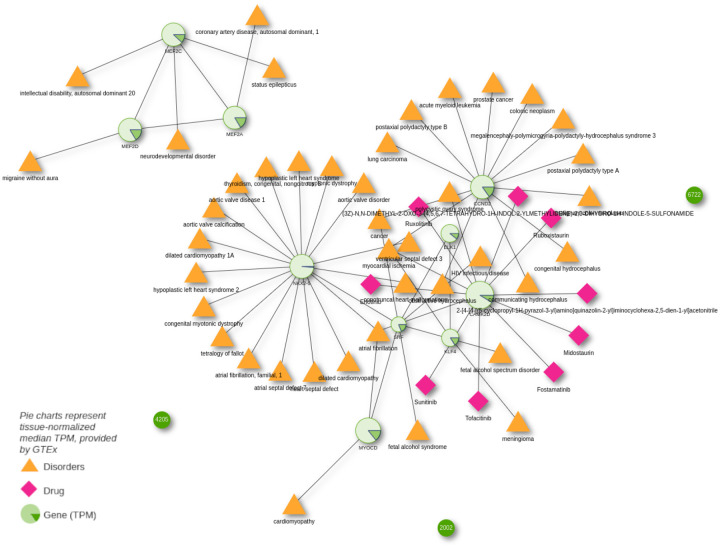
Participants of WikiPathway WP1991 displayed in Drugst.One. Adjacent diseases and drugs are enabled, as well as diseases linked to drugs targeting this smooth muscle cell proliferation and differentiation pathway. Normalized median expression values for ‘Artery - Aorta’ are overlaid as pie charts, where 360° represent the maximum observed transcripts per million (TPM) in the selected tissue and all other TPMs are exponentially scaled.

WikiPathway WP1991 describes the mechanism behind smooth muscle cell (SMC) differentiation and proliferation. The WikiPathways web interface now incorporates a button to export the pathway genes into Drugst.One (using the magnifier glass in the table showing the proteins participating in the pathway, state 03.07.23) and visualizing their interactions with drugs in Drugst.One directly. To gain a general overview of the complications (symptoms, comorbidities, etc.) associated with dysfunctional SMC development and their drivers, Drugst.One allows for extending the WikiPathways-exported network by the corresponding disorders and their associated pathway genes. Several disease nodes appear in the network, mainly representing various cardiovascular disorders (CVDs), e.g., cardiomyopathy, coronary artery disease, and aortic valve (Figure 3). The importance of SMCs for proper vascular functionality [34,35] and thus to atherosclerosis, hypertension, myocardial infarction, and other cardiovascular diseases was reported before [36–38]. An isolated subnetwork community of proteins is formed by the three myocyte enhancer factors *MEF2A*, *MEF2C*, and *MEF2D*. Besides their obvious cardiovascular implications, these factors play a role in neurological processes [39]. An impact of SMCs on status epilepticus was shown in mouse models [40], and a connection between migraine and SMC dysfunction was suggested as well [41,42].

Drugst.One allows for the projection of (gene) expression data from GTEx on the proteins in the network. The relative expression of these genes appears to be quite high in arteries and organs that have to perform physical motion, like heart, lung, bladder, and skeletal muscles, but with observable fluctuations in the relative expression of genes like *CCND2*.

With one mouse click, we import drug target information for drug repurposing candidate prediction. Despite SMCs relation to cardiovascular diseases, no corresponding CVD drugs have been identified. Mainly anti-cancer drugs (e.g., sunitinib, erlotinib, midostaurin, and ruxolitinib) targeting calcium/calmodulin-dependent protein kinase II delta (*CAMK2D)*, which is associated with cancer growth [43], are found. Notably, however, *CAMK2D* also plays a role in calcium signaling, which is essential for the upkeep of SMC function [44,45]. Hence, this may explain the observed cardiovascular side effects of *CAMK2D*-targeting drugs. According to SIDER [46], sunitinib may cause hypertensive symptoms and corresponding studies suggest that midostaurin has cardiotoxic effects [47].

Algorithms integrated in Drugst.One can extend the search space by looking for indirectly connected drugs. The selection menu offers a function to automatically add all displayed proteins to the selection, serving as the starting point (seeds) of subsequent searches. The harmonic centrality algorithm (see Supplement 3.2.3) was used to extend the network by the ten drugs with the highest score, including indirectly (transitively) connected drugs from the NeDRex database. Through this search, the tyrosine kinase inhibitor nintedanib, which has shown promising effects in pulmonary arterial smooth muscle cells and intestinal smooth muscle cells [48,49], can be identified.

This shows the identification potential of mechanism-associated drugs through the network-based drug repurposing functions Drugst.One incorporates. Whereas before only drugs primarily used in cancer were present through direct association with SMC pathway participants, Drugst.One suggested more relevant options for this context.

## Discussion

Biomedical research generates a wealth of data that could inform the development of novel therapies or treatments. However, despite this potential, a significant portion of the analyses conducted in this field fail to translate into clinical trials, leading to major issues in the effectiveness of public health research [50]. To this end, Drugst.One has the potential to help transform specifically omics-based research results into actionable hypotheses with potential clinical impact. Drugst.One offers a community-driven solution to streamline the knowledge distributed over many online resources for multi-omics analyses and other biomedical tools [51] to turn the results of biomedical analyses into concrete candidate drug targets and drug repurposing hypotheses. Still, we emphasize that the drug target and drug repurposing predictions are merely candidates and supervision with expert knowledge is still required before experimental validation. Drugst.One delivers explainable indications based on established biological data like expression and known disease associations or drug indications, however, the interpretation of their application in the case-specific context is up to the user. Therefore, we designed Drugst.One to be operated with maximal transparency and allow optional user input for every step of the analysis.

With the infrastructure and the resources being provided, Drugst.One helps to find a community-wide solution for standardization and streamlining the visualization of explainable disease modules and their pharmacological implications. Drugst.One provides various interfaces to be highly accessible and customizable by all members of the community while maintaining up-to-date database information and network analysis algorithms. Smooth integration into most biomedical websites and tools is confirmed by 21 resources already integrating Drugst.One. For future developers who wish to customize Drugst.One before its integration, an interactive web interface provides copy-paste-able code for customized plugin integration with their own website. An endpoint for developers who want to link out from any of their websites, apps, or command line tools is provided by Drugst.One as well.

Drugst.One complies with community standards regarding data management as defined by the FAIRness principles [52]. Download links for any data shown in Drugst.One are provided at any step, whether it is a table with drug target and drug candidates or the visualized network with all activated extensions like expression information. Export to current community standards is supported via exporting compatible .graphml files, which can be loaded directly into, e.g., Cytoscape [13]. To further increase reproducibility and interoperability, concrete plans to implement save and export functions of Drugst.One networks to NDEx [53,54] are made.

In summary, Drugst.One offers an important service to the systems medicine research community to tackle the widely recurring problem of web-based disease mechanism mining and drug repurposing candidate prediction by capturing the results of biomedical assays.

## Supplementary Material

1

## Figures and Tables

**Table 1.** Systems medicine tools that integrate Drugst.One listed in alphabetical order. Options are ‘Link-out’, referring to a URL based redirect from the tool or website to the Drugst.One standalone page, ‘Plugin’, referring to the integration of the javascript-based plugin into the web tool, and programmatic access using the ‘Python package’.

Tool	URL	Tool Description	Integration	Integration status
BiCoN [8]	https://exbio.wzw.tum.de/bicon/	Network-constrained patient stratification through biclustering	Plugin	Done
DOMINO [21]	http://domino.cs.tau.ac.il/	Active module identification with improved empirical validation	Link-out	In progress
G-Browser	https://exbio.wzw.tum.de/genome-browser/	An enhanced genome browser plugin that seamlessly integrates data sources and functions for genetics research.	Plugin	Done
GraphFusion	https://github.com/CarlosJesusGH/GraphFusion	An intuitive web-based graph analytics, fusion, and visualization tool	Plugin	Done
GraphSimViz [22]	https://graphsimviz.net/	Visualization of diseasomes, drugomes, and drug-disease networks	Plugin	Done
HitSeekR [23]	https://exbio.wzw.tum.de/hitseekr/	User-friendly tool for drug (target) identification in high-throughput screening	Plugin	Done
Interactive Enrichment Analysis [24]	https://github.com/gladstone-institutes/Interactive-Enrichment-Analysis/	Enrichment analysis on multiple public datasets	Link-out	In progress
mirDIP [16]	https://ophid.utoronto.ca/mirDIP/	Integrated microRNA-target data integration portal	Plugin	Done
NAViGaTOR [25]	https://ophid.utoronto.ca/navigator/	Network visualization and analysis software	Link-out	In progress
NDEx IQuery [20]	https://www.ndexbio.org/iauery/	Web tool for pathway and network-based gene set analysis	Plugin	Planned
NeEDL - Epistasis Disease Atlas	https://epistasis-disease-atlas.com	Web resource to visualize, investigate, and interpret higher-order genetic interactions of single nucleotide polymorphisms in 18 human heritable diseases.	Link-out	Done
NeEDL - R Shiny App	https://hub.docker.com/r/bigdatainbiomedicine/needl	R shiny app to visualize, investigate, and interpret higher-order genetic interactions of single nucleotide polymorphisms on locally computed datasets.	Plugin	Done
openPIP [26]	https://github.com/BaderLab/openPIP	Open platform to store and retrieve protein-protein interaction datasets.	Link-out	Done
pathDIP [27]	https://ophid.utoronto.ca/pathDIP	Integrated pathway database and pathway enrichment analysis portal	Plugin	In progress
Pathway Figure OCR [28]	https://pfocr.wikipathways.org	Platform for browsing pathway information extracted from published figures.	Link-out	Done
ProHarMeD	https://proharmed.zbh.uni-hamburg.de/	Closing the gap between (harmonized) proteomics results and mechanotyping / drug repurposing	Plugin	Done
ROBUST-Web [29]	https://robust-web.net/	ROBUST is a disease module identification tool.	Plugin	Done
SCANet	https://pypi.org/proiect/scanet/	SCANet is an all-in-one package for single-cell profiling covering the whole differential mechanotyping workflow, from inference of trait/cell-type-specific gene co-expression modules to mechanistic drug repurposing candidate prediction.	Python package	Done
Seed Connector Algorithm [30]	https://github.com/bwh784/SCA	Identification of network modules by adding a minimal number of edges between the seed nodes.	Link-out	Done
UnPaSt	https://unpast.zbh.uni-hamburg.de	Visualizer and context explorer for unsupervised expression data bicluster results.	Plugin	Done
WikiPathways [17]	https://wikipathways.org	Platform for browsing and visualizing pathways.	Link-out	Done
