# Supplementary material for: Drugst.One - A plug-and-play solution for online systems medicine and network-based drug repurposing
Source: ArXiv. 2023 Jul 4:arXiv:2305.15453v2. Originally published 2023 May 24. Preprint. [Version 2] (PMC10274948)
Supplement: 1 [file NIHPP2305.15453V2-supplement-1.pdf]

Supplementary information

1. Drugst.One ecosystem

The Drugst.One ecosystem consists of the Drugst.One plugin for developers and a content delivery system (CDS) to distribute it, a website, a backend server, and a Python package (Figure 2). Any communication between Drugst.One components is SSL encrypted using HTTPS. We use GitHub for versioning of plugin and backend as well as code example repositories, all of which can be found here: https://github.com/drugst-one. For the Drugst.One website, the CDN server, CI/CD pipelines and container registries we, for now, use a GitLab instance hosted by the RRZ, the computing center of the University of Hamburg.

1.1 Plugin

The Drugst.One web plugin visualizes and enriches protein-protein interaction (PPI) networks (Figure S1). Given a set of gene or protein identifiers, or small networks, interaction information can be completed automatically from the Drugst.One database and the users of the website can explore the nodes in their network context. With one click, transitive connections between otherwise unconnected proteins within the interactome can be identified or first-neighbor diseases and drugs can be added to the network. Further, tissue-specific expression of the proteins can be highlighted directly in the network.

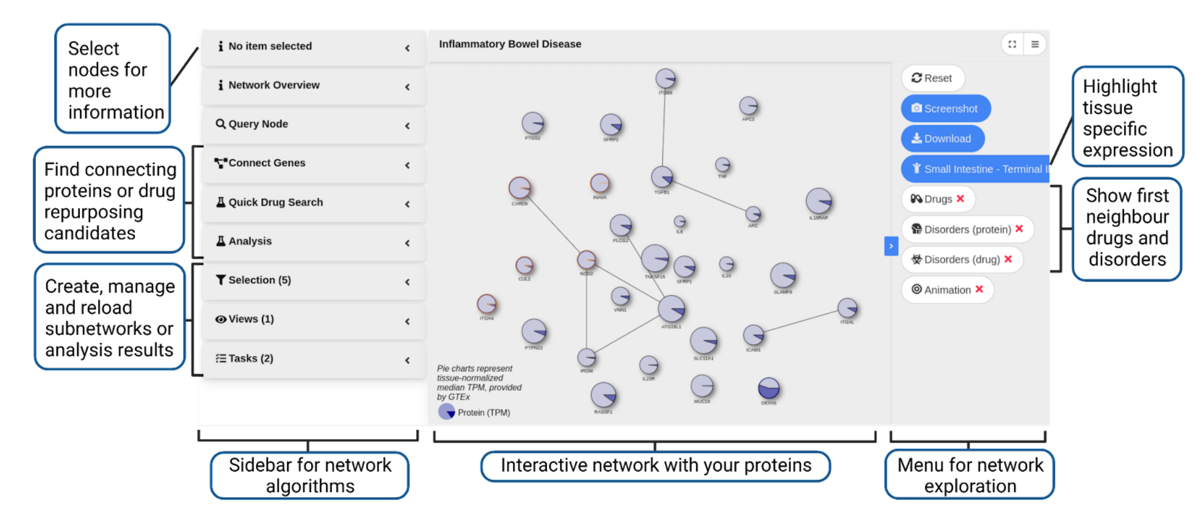

Figure S1: The interface of the Drugst.One plugin consists of a sidebar with network information and options for drug repurposing algorithms (left), an interactive network visualization (center), and a menu with functions to modify the network (right). Individual components and positions of these elements can be configured. Visualized are the 30 genes from the inflammatory bowel disease use case (section 4 in Supplementary Notes).

Besides exploring the loaded genes or proteins, the plugin can be used to generate drug repurposing hypotheses. Using the explorative functionalities such as highlighting proteins based on their expression in tissues, input proteins for analysis tasks can be selected. Leveraging the power of specialized network algorithms, disease modules can be identified to find additional drug targets within the same genetic context and drug repurposing candidates can be prioritized (Figure S2). Analysis results are stored for future access and can be loaded directly into the interface where a network visualization helps to understand the relation of the result to the input nodes. New analyses can be started on the original network or based on previous analysis results, supporting an iterative approach to a transparent and user-driven drug repurposing.

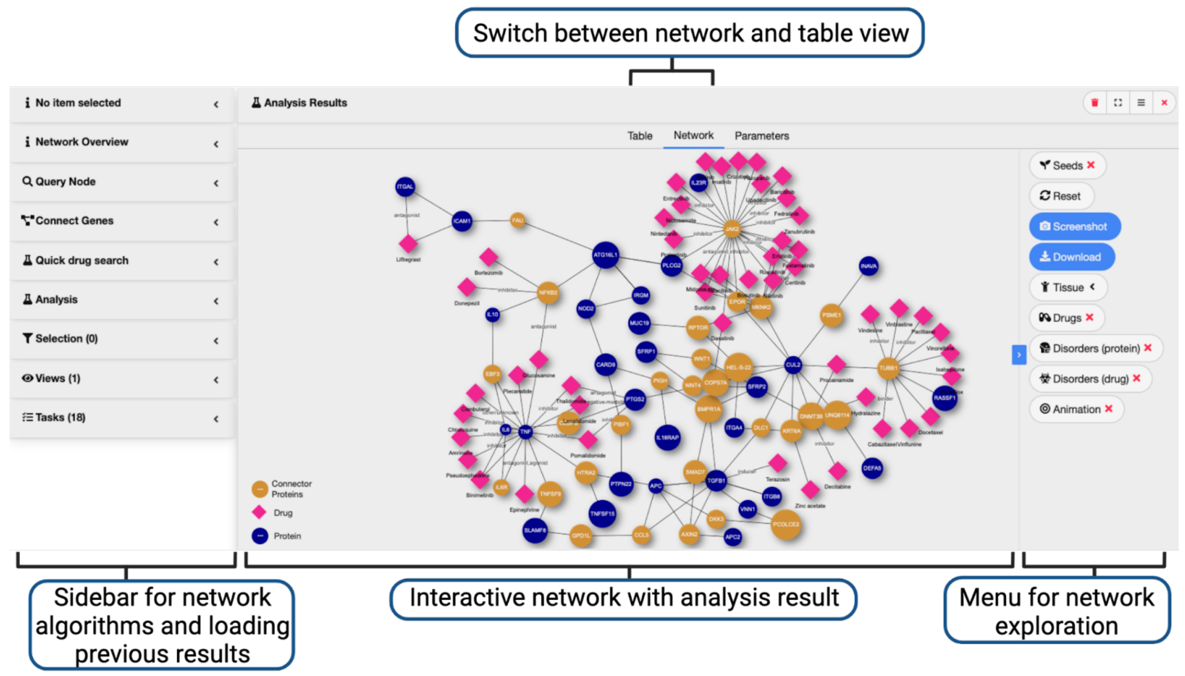

Figure S2: The results of drug target and drug repurposing candidates search for the inflammatory bowel disease use case (Supplements 4) are represented in the Drugst.One plugin as a network. A ‘Quick Drug Search’ was executed on all proteins; hence, a Multi-Steiner tree was used to connect the genes and a harmonic centrality algorithm to identify putative drug candidates.

Despite all these features the plugin remains completely serverless for hosting websites as all computations take place on the Drugst.One servers. The customizable plugin consists of a JavaScript-based web component, which can be fitted to the needs of the host website through JSON-formatted configuration strings. Functions, buttons, colors, and dimensions of the component can be adjusted to seamlessly blend in with the rest of the page. Style encapsulation guarantees no interference with styles from the host webpage. Lastly, the Drugst.One plugin is lightweight, no heavy libraries will be added to the host's webpage to minimize loading times.

The latest Drugst.One plugin (at submission v1.1.19) is developed in Angular.js 14 with TypeScript 4.4.4 and compiled and packaged with npm 8.15.0 and node 16.17.1 into

drugstone.js and drugstone.css files. For network visualization, vis.js (v9.1.6) is used and screenshot support is provided through dom-to-image-cross-origin (v.2.6.7).

1.2 Content delivery system

To enable the easy use and distribution of the plugin, we set up a simple content delivery system (CDS) to account for a large number of simultaneously loaded plugin instances on multiple hosting web pages. The CDS manages the plugin versions by maintaining all builds of previous plugin versions that can be accessed through fixed version identifiers. The latest stable release is tagged 'latest' and will be updated upon a new stable release. Unstable development versions can be tested using versions tagged as release candidates ('-rc'). The most current development version can be accessed through the tag 'nightly'. A list of all available plugin versions is generated after each build and is available at https://cdn.drugst.one/.

1.3 Website

The Drugst.One website (https://drugst.one/) serves as a documentation and exploration environment for the Drugst.One plugin. Many examples in the documentation and tutorial videos guide users through the individual features of the Drugst.One plugin as well as its integration.

The Drugst.One playground provides a graphical interface to conveniently generate custom "copy-and-paste" code snippets incorporating style and configuration choices made by the user. Further, a standalone version of the Drugst.One plugin is integrated into the website and can be accessed and modified by passing parameters using GET or POST HTTP requests, allowing developers to use the plugin without having to host a web service or incorporate the plugin in a webpage. Additionally, to facilitate and initiate the development of novel web tools presenting biomedical networks, it provides a website application template with the Drugst.One plugin.

1.4 Server

The Drugst.One backend server consists of a Django API (v3.2.19) connected to a PostgreSQL (v14) database in combination with a redis broker and worker (v7.0.11) and a Celery scheduler (v5.2.7). The backend interacts closely with the plugin, instantly returning information to the loaded proteins and adding the interactions between the proteins to create the PPI network. It further handles the asynchronous execution of the network algorithms in the analysis tasks by starting separate jobs on the redis server, allowing it to handle a large number of jobs in parallel or queue them accounting for the workload of Drugst.One instances on multiple websites. Most network algorithms (Supplement 3.2) are implemented with graph-tool (version 2.55), a performant C++-based network library for Python, and pre-processed network files, resulting in fast execution times of usually less than 30 seconds.

1.5 Python package

Programmatic access is supported using Python 3.6 and newer. The Python package (https://pypi.org/project/drugstone/) can be installed using pip (‘pip install drugstone’) and supports the main functionalities of the plugin, including fetching protein information, PPIs, and conducting drug repurposing analyses. With the Drugst.One python package, a larger number of tasks can be executed in an automated fashion, then form the GUI of the plugin, empowering developers to integrate Dugst.One into custom workflows of their own programs.

2. Integration

2.1 Plugin

Developers can add the plugin to their own website using JavaScript. This may help to visualize any results containing a list of proteins or PPIs and to add the Drugst.One functionalities. Integration is done in three steps. Firstly, the Drugst.One libraries need to be loaded with the website by integrating the import statement into the head tag of your website. It is possible to define a specific version identifier to use a static version of the plugin or to use the tag ‘latest’ to automatically update to the latest stable version upon a new release.

```
<head>
   <script src="https://cdn.drugst.one/latest/drugstone.js"></script>
   <link rel="stylesheet"
href="https://cdn.drugst.one/latest/styles.css">
</head>
```

Secondly, the Drugst.One component needs to be placed on the website. After loading the Drugst.One libraries, the html tag ‘drugst-one’ becomes available. The position of the tag defines the position of the component on a webpage.

```
<drugst-one id='drugstone-component-id'></drugst-one>
```

Lastly, the component can be configured by passing options as JSON strings to the three attributes ‘groups’, ‘config’, and ‘network’. The ‘network’ parameter can accept lists of nodes and optionally edges to construct the network. Each node and edge in the network is assigned to a node and edge group, respectively, which defines the styles of each member. Additionally, groups are assigned a group name that all its members inherit, e.g. ‘protein’ or ‘drug’. Individual nodes and edges may receive individual styles to highlight them which will override the group styles. Because the network is based on vis.js, all node and edge attributes used in vis.js are applicable, including e.g. directed edge styles. Settings regarding the component itself e.g. to add or remove certain features, or which datasets to use may be passed to the ‘config’ parameter.

```
<drugst-one
groups='{"nodeGroups":{"Protein":{"type":"Protein","color":"#ff881f","fo
nt":{"color":"#ffffff"},"groupName":"Protein","shape":"ellipse"}},
"edgeGroups":{"PPI":{"color":"#111111", "groupName":"PPI"}}}'
   config='{"identifier":"symbol","title":"Breast cancer example
network"}'
   network='{"nodes":[{"id":"BRCA1","label":"BRCA1", "group":
"Protein"}, {"id":"BRCA2","label":"BRCA2", "group": "Protein"}],
"edges": [{"from":"BRCA1", "to":"BRCA2"}]}'>
  </drugst-one>
```

The Drugst.One plugin adapts to any changes of the options immediately, allowing developers to add features like buttons, toggles, or selections to e.g. change or adjust the network at runtime.

Custom styling of the component can be achieved by setting global CSS parameters on the website. Through the prefix ‘drgstn’ it is ensured that CSS variables will not randomly collide with other style variables.

```
:root {
   --drgstn-primary:#347eee;
   --drgstn-secondary:#2e42f2;
   --drgstn-success:#48C774;
   --drgstn-warning:#ffdd00;
   --drgstn-danger:#ff2744;
   --drgstn-background:#f8f9fa;
   --drgstn-panel:#ffffff;
   --drgstn-info:#61c43d;
   --drgstn-text-primary:#151515;
   --drgstn-text-secondary:#eeeeee;
   --drgstn-border:rgba(0, 0, 0, 0.2);
   --drgstn-tooltip:rgba(74,74,74,0.9);
   --drgstn-panel-secondary:#FFFFFF;
   --drgstn-height:600px;
   --drgstn-font-family:Helvetica Neue, sans-serif;
}
```

2.2 Standalone

In cases where it is not desired or possible to integrate the Drugst.One component to a website, e.g., because a tool has no website, the version hosted at the Drugst.One website is accessible using HTTP requests. When working with small networks and little customization of the plugin is required, it is sufficient to encode the parameters in GET requests.

```
https://drugst.one?nodes=PTEN,TP53&edges=PTEN%20TP53&autofillEdges=false
```

For the GET-based configuration, only selected parameters are available (https://drugst.one/doc#standalone_api).
Drugst.One buttons are provided to facilitate the integration.

```
<link rel="stylesheet"
href="https://cdn.drugst.one/libs/drugstone-buttons/0.0.1/drugstone-butt
ons.min.css">

<a class="drugstone-button drugstone-grey"
href="https://drugst.one/standalone?nodes=PTEN,TP53,BRCA2&autofillEdges=
true&activateNetworkMenuButtonAdjacentDrugs=true&interactionDrugProtein=
NeDRex&licensedDatasets=true" target="_blank">Drugst.One</a>
```

To pass more network data or extensive configuration parameters that are otherwise not available or exceed the URL limit of 2048 characters, POST requests can be used. An API endpoint expects the same options as the plugin and returns an identifier with which the network can be loaded using a GET request (i.e. an URL):

```
(Send options to API and GET identifier)
let networkID = post(
   'https://api.drugst.one/create_network',
   {
       network: {nodes: [...], edges: [...]},
       groups: {...}
       config: {...}
   }
)
```

```
(Load configuration in Drugst.One standalone with a network identifier)
https://drugst.one?id=<networkID>
```

3. Methods

3.1 Data integration

An essential contribution of Drugst.One is the integration of multiple data sources that can be selected to add information to visualized data. Basic entities that are considered in Drugst.One are proteins/genes, drugs, and diseases. The available ID spaces for gene or protein entities are HGNC [1], UniProt [2], Ensemble [3], and Entrez [4]. For drugs Drugst.One uses DrugBank and for disorders MONDO [5] identifiers. To describe links between the different entities Drugst.One integrates four different relational layer types, namely protein-protein, protein-drug, protein-disorder, and drug-disorder data, derived from multiple different data sources (Supplementary Table 1). Another distinction between static and updating datasets can be made. Using the secondary database NeDRexDB [6], which is updated on a weekly basis, any data imported from it is automatically updated weekly using celery-beat as a scheduler. The NeDRex datasets for protein-protein and drug-target interaction data represent a combination of all individual data sources. Data that is not available in NeDRex do not receive regular updates. Some data sources have restrictive reuse licenses attached, e.g., for use in a commercial scenario. In Drugst.One, we provide both, licensed and openly available datasets, but the access to licensed data has to be unlocked with a configuration parameter. At the time of publication, the following datasets and their respective the end-user license agreements (EULA) are available in the Drugst.One plugin menu:

Source	Version	Layers	Licensed
APID [7]	January 2019	Protein-Protein	no
BioGRID* [8]	2023-07-03	Protein-Protein	no
ChEMBL [9]	27	Protein-Drug	no
CTD* [10]	2023-07-03	Drug-Disorder	no
DGIdb [11]	4.2.0	Protein-Drug	no
DisGeNET*[12]	2023-07-03	Protein-Disorder	no
DrugBank [13]	5.1.8	Drug-Disorder	yes
DrugBank* [13]	2023-07-02	Protein-Drug	yes
DrugCentral* [14]	2023-07-03	Protein-Drug, Drug-Disorder	no
GTEx [15]	v8	Tissue Expression	no
IID* [16]	2023-07-03	Protein-Protein	no
IntAct* [17]	2023-07-03	Protein-Protein	no
NeDRex [6]	2.10.0	Protein-Protein, Protein-Drug, Protein-Disorder, Drug-Disorder	yes
NeDRex [6]	2.10.0	Protein-Protein, Protein-Drug, Protein-Disorder, Drug-Disorder	no
OMIM* [18]	27-12-2022	Protein-Disorder	yes
STRING [19]	11.0	Protein-Protein	no

Supplementary Table 1. List of association types and source databases stored in the Drugst.One data warehouse. The version refers to the latest state before submission. *Databases with an asterisk are integrated as a part of NeDRexDB.

3.2 Algorithms

Seven network mining algorithms are implemented in Drugst.One for module identification and/or drug prioritization. To keep the plugin lightweight and easy to use, algorithms were deliberately chosen due to their focus on basic network properties like degree centrality and network proximity. Depending on the type of network, e.g. sparsely or densely connected, users can try out different approaches to explore the search space of related drug targets

and drug repurposing candidates. While all of the algorithms share some general parameters (Supplement 3.2.1), some of the algorithms may offer additional settings (Supplement 3.2.2 - 3.2.9).

3.2.1 General parameters

For each algorithm the following options exist:

- Result Size. The number of returned nodes (drug targets or drugs)
- Maximum Degree. Option to filter out hub genes. If set to > 0, genes with a network degree in the complete gene-drug interaction network above this threshold will be excluded.
- Hub Penalty. Penalizes genes with a large degree in the network.
- Filter Edges. If set, only the shortest paths to the drug target or drug nodes will be displayed in the resulting network. Otherwise, all found pathways will be shown.

3.2.2 Betweenness centrality

Betweenness is obtained by finding the shortest paths for each pair of nodes in the network and assessing the number of shortest paths that pass through a particular node, such that a measure of the centrality of a node in a network global context is received. Betweenness centrality has been established as a common measurement in network biological applications [20] and is especially practical in finding communities in large networks [21]. In Drugst.One, betweenness is based on the shortest paths between the seed nodes only and can be used to find drug targets with maximized connectivity to all seeds.

3.2.3 Harmonic centrality

Harmonic centrality (C_h) measurement can be described as the average shortest distance from each node to all other nodes in a network. This measurement is the equivalent of harmonic centrality for disconnected graphs. Formally speaking, it can be annotated as

$$C_h = \sum_{y \neq x}^{y} \left(\frac{1}{dist(x,y)} \right)$$

where x is a given node and $\frac{1}{dist(x,y)} = 0$ if $dist(x,y) = \infty$ [22]. The closer a node is to other nodes, the higher the score. It has already been proven successful in a number of biological network problems for instance with metabolic or PPI networks [23,24].

3.2.4 Degree centrality

Degree centrality (C_d) measurement is obtained by ranking the nodes in a network based on their degree, which is defined as the number of neighbors a node has divided by the total number of nodes in the network. It can be described as

$C_d(x) = deg(x)$

where x is a given node and $deg(x)$ is its degree. While it is a commonly used network analysis technique, it most importantly has been shown useful in the identification of essential proteins in PPI networks [25,26]. Thus, it is a simple approach for classifying the network-related importance of a particular protein. In Drugst.One, it can be used to discover valuable drug targets or drugs, based on the seed selection given by the user. However, ranking by node degree is prone to introduce research bias.

3.2.5 KeyPathwayMiner

KeyPathwayMiner (KPM) is an online tool developed by Alcaraz et al. for pathway enrichment analysis [27]. Users can utilize KPM for their drug target search by selecting seed genes from the network and letting KPM find an interaction network of genes spanned by the seed genes. The resulting genes are functionally related to the seed nodes and therefore are suitable drug target candidates. Only one parameter k has to be set by the user, which defines the amount of permitted intermediate nodes that are neither part of the seed nodes nor the common pathway.

- Additional proteins k: Number of intermediate nodes allowed between the seed nodes.

3.2.6 Multi-Steiner tree

The Multi-Steiner tree algorithm [28] approximates the minimum spanning tree connecting the seed nodes in a reasonable time. The implementation is adopted from Ahmed et al. [29]. It can be used to create a minimum spanning subnetwork between user-selected seed nodes, which happen to be central interaction partners between the seed nodes and thus represent favorable drug targets. The user can specify the number of Steiner trees computed to approximate a minimum spanning tree, and the tolerance indicating how much the subsequent trees may increase the number of edges a higher number of Steiner trees leads to more variations at the cost of a longer runtime.

3.2.7 Network proximity

As introduced by Guney et al. [29], network proximity is the average length of shortest paths from drug target nodes to all of the user-selected seed nodes. The algorithm then computes a statistical significance score compared against random expectation. This algorithm was adopted in Drugst.One so that best-scored drugs are returned to the user as candidate drugs.

3.2.8 TrustRank

TrustRank [30] is based on the same concepts as the Google PageRank algorithm and harmonic centrality [31,32]. A trust score is propagated through the network starting at the

seeds, damping the score based on the distance traveled. The user can set the damping factor in a range from 0-1, with a higher damping factor causing the propagation to either stop at nodes in close proximity or in larger portions of the network. In Drugst.One, TrustRank is used to identify putative drug targets as well as drug candidates.

- Damping Factor: Correlates with the distance a trust score propagates through the network. The larger the factor, the larger the proportion of the network that is considered.

3.2.9 Algorithm applications

**Name**	**Drug target search**	**Drug search**
Betweenness centrality	yes	no
Harmonic centrality	yes	yes
Degree centrality	yes	yes
KeyPathwayMiner	yes	no
Multi-Steiner tree	yes	no
Network proximity	no	yes
TrustRank	yes	yes

Supplementary Table S2. An overview of all integrated algorithms regarding their availability in drug target and drug search functions.

4. Use Case: IBD

4.1 Repurposing of JAK inhibitors against IBD

A central aspect of Drugst.One is the focus on *in-silico* drug repurposing candidate prediction. To this end, we replicate an exemplary repurposing case study for inflammatory bowel disease (IBD).

Sadegh et al. [6] identified fostamatinib, ruxolitinib, and imatinib for application in IBD by starting from 30 seed genes associated with IBD according to DisGeNET [12] and OMIM [18] (Supplementary Figure S1, Table S3). Multi-Steiner tree (MuST) [28] was applied to connect the seed genes in the network and the closeness centrality (CC) algorithm was employed to identify drug repurposing candidates (Supplementary Figure S2).

To reproduce this example case in Drugst.One (see Table S3), we loaded the 30 seed genes associated with IBD into Drugst.One. As protein-protein and drug-target interaction datasets the licensed NeDRex versions were used and we executed a 'Quick Drug Search', consisting of a 'Drug target search' using MuST (trees=5, tolerance=5, hub penalty=0.5) to connect all seeds. Drugs were ranked using harmonic centrality, being the closest to CC used in the original paper, and the top 50 drugs were chosen as the most promising candidates. In the referenced paper, the authors identified fostamatinib, ruxolitinib, and imatinib on ranks 1, 5, and 12 respectively as drugs that have literature support for being relevant for IBD. With Drugst.One, the same drugs are found at ranks 4, 4, and 9, (Supplementary Tabe S5). The difference might stem from small variations in the interactome and drug-target data or the use of harmonic centrality instead of closeness centrality. The drugs target *JAK2*, a gene added by MuST, with ruxolitinib and fostamatinib being known JAK inhibitors (JAKi), and their potential for IBD treatment is currently under investigation [33]. Further, they inhibit *MKNK2*, another gene identified by MuST and investigated for its role in different types of colitis [34]. JAKis interact with *MAPK*, a gene well-known for its role in IBD [35,36], with which the observed effect of a dysregulated *MKNK2* might be explained. Tofacitinib, another JAKi and ranked second by HC, has the same targets as fostamatinib and ruxolitinib and is subject to studies investigating beneficial effects in IBD [37,38].

Dasatinib, the tyrosine kinase inhibitor on rank one, is known to induce ulcerative colitis, part of the IBD umbrella, in some patients [39,40]. Even though this drug does not have the desired effect, it is directly associated with the targeted disease and its pathways and may lead to the identification of impactful targets.

Rank two is shared by three immunomodulatory drugs thalidomide, pomalidomide and lenalidomide, as well as glucosamine. Thalidomide has known indications for ulcerative colitis, a subtype of IBD, while also lenalidomide and pomalidomide showed protective effects against IBD in mouse models [41]. Lopez-Millan et al. identified lenalidomide as the more potent option, suggesting its usage as a therapeutic drug against inflammatory diseases. There is evidence of beneficial effects of these immunomodulators in human IBD, but their use in clinical practice is under discussion due to severe adverse side effects [42,43].

Binimetinib is ranked third, which is mainly used as an anti-cancer drug. In this application, inflammatory colitis was observed as an adverse side effect [44], hinting towards a cause-effect relationship that can be studied further to research the mechanistic origins of

IBD.
On rank four, together with ruxolitinib and fostamatinib, other inhibitors with the same targets (*MKNK2* and *JAK2*) are found, namely tofacitinib (FDA-approved for ulcerative colitis), sunitinib, midostaurin, erlotinib, and ceritinib.
On ranks two to five, tumor necrosis factor-α (*TNF*) inhibitors are found. *TNF* is a known target to treat IBD [45,46] and thus effects of the drugs chloroquine on ulcerative colitis [47] and plecanatide on colitis symptoms in mice [48,49]. Further, epinephrine, pseudoephedrine, and clenbuterol share rank five, all have *TNF*-inhibiting effects [50–52] and appear to have general anti-inflammatory effects [53], most likely induced by downregulated or inhibited *IL-6* expression [52,54].
Rank six terazosin, a drug inhibiting *TGFB1*, whose dysregulation is closely linked to IBD [55].
In summary, with Drugst.One we were not only able to re-identify three promising repurposing candidates from a previous study for IBD, but show that the first 5 ranks (that includes 20 drugs) contain valid candidates or already approved drugs for IBD treatment, including hypotheses about their molecular relationship with IBD.

The following 30 seeds are IBD-associated genes that were used by Sadegh et al. [6] to build their use case.

ATG16L1	ICAM1	TNF	SFRP2	APC2
IL10	CUL2	SFRP1	TNFSF15	ITGA4
DEFA5	MUC19	SLAMF8	APC	IL6
INAVA	CARD9	ITGB8	IL23R	NOD2
RASSF1	SLC11A1	IL18RAP	TGFB1	IRGM
PLCG2	PTPN22	PTGS2	VNN1	ITGAL

Supplementary Table S3. List of the 30 IBD-associated genes used by Sadegh et al. [6] in their drug repurposing case study. This list serves as input for an example use case highlighting the potential of Drugst.One.
The parameters that can be used to replicate the use case, may be found in table S4.

Drugst.One	
Protein-Protein interaction dataset	NeDRex (licensed)
Drug-Protein (target) interaction dataset	NeDRex (licensed)
**Drug target search**	
Algorithm	MuST
Number of Steiner trees	5 (default)
Tolerance for trees	5
Hub penalty	0.5
**Drug search**	
Algorithm	Harmonic centrality
Result size	50

Supplementary Table S4. The Drug-Protein, as well as the Protein-Protein datasets, used for the IBD drug repurposing use case, were set to the most complete one (NeDRex - licensed). For reproducibility, the exact parameters used in drug target and drug identification steps are listed.

**Drug**	**Score**	**Rank**
Dasatinib	1	1
Lenalidomide	0.9882636549013167	2
Thalidomide	0.9882636549013167	2
Glucosamine	0.9882636549013167	2
Pomalidomide	0.9882636549013167	2
Binimetinib	0.9824981860163579	3
Erlotinib	0.9600936780691096	4
Sunitinib	0.9600936780691096	4
Ruxolitinib	0.9600936780691096	4
Tofacitinib	0.9600936780691096	4
Midostaurin	0.9600936780691096	4
Fostamatinib	0.9600936780691096	4
Ceritinib	0.9600936780691096	4
Chloroquine	0.9600936780691095	5
Epinephrine	0.9600936780691095	5
Clenbuterol	0.9600936780691095	5
Pseudoephedrine	0.9600936780691095	5
Amrinone	0.9600936780691095	5
Plecanatide	0.9600936780691095	5
Terazosin	0.9492702822172273	6
Bortezomib	0.9334851363762997	7
Donepezil	0.9334851363762997	7
Lifitegrast	0.9283394387790588	8
Pralsetinib	0.9232501599628847	9
Nilotinib	0.9232501599628847	9
Niclosamide	0.9232501599628847	9
Fedratinib	0.9232501599628847	9
Imatinib	0.9232501599628847	9
Bosutinib	0.9232501599628847	9
Crizotinib	0.9232501599628847	9
Nintedanib	0.9232501599628847	9
Upadacitinib	0.9232501599628847	9
Entrectinib	0.9232501599628847	9

Pazopanib	0.9232501599628847	9
Zanubrutinib	0.9232501599628847	9
Axitinib	0.9232501599628847	9
Baricitinib	0.9232501599628847	9
Procainamide	0.9083117074801125	10
Decitabine	0.9083117074801125	10
Hydralazine	0.9083117074801125	10
Zinc acetate	0.9083117074801123	11
Vinflunine	0.8986184380435127	12
Ixabepilone	0.8986184380435127	12
Vinblastine	0.8986184380435127	12
Podofilox	0.8986184380435127	12
Paclitaxel	0.8986184380435127	12
Docetaxel	0.8986184380435127	12
Cabazitaxel	0.8986184380435127	12
Vindesine	0.8986184380435127	12
Vinorelbine	0.8986184380435127	12

Supplementary Table S5. The top 50 drugs resulting from the 'Quick Drug Search' in the IBD drug repurposing use case. The search was conducted on the NeDRex dataset (licensed). Listed are the drugs with their respective score (normalized) as returned by algorithm as well as the assigned rank.

4.2 Drug candidate identification and mechanism mining through microRNA targets

The database of human microRNA (miRNA) target predictions, mirDIP (version 5.3.0.1, database version 5.2.3.1) [56], is a resource for miRNA-based regulation information. mirDIP allows the identification of gene regulation through miRNAs while avoiding a prediction bias. Its unidirectional search function can be used to either find miRNAs targeting a given set of genes or to retrieve the set of targeted genes given a set of miRNAs. In both cases, mirDIP now offers the option to visualize and explore the used or found genes using the Drugst.One plugin.

We took 30 IBD-associated genes from Sadegh et al. [6] (Supplementary Table S3) as mirDIP input to identify all known or predicted miRNA regulators ('miRNA-gene matrix' -> 'Search gene symbols'). Three microRNAs (*hsa-miR-142-3p*, *hsa-miR-3942-5p*, and *hsa-miR-574-3p*) were deemed to be main regulators of IBD genes because they each target three IBD-associated genes. Interestingly, *hsa-miR-142-3p* levels have been found to be elevated in the saliva of ulcerative colitis (UC) patients [57]. For these microRNAs, we identified the targets using mirDIP unidirectional search and loaded the list of all targeted genes into the Drugst.One plugin to identify relevant drugs and disorders (Figure S3).

Using the first neighbor drug and first neighbor drug-disease association annotation in Drugst.One a number of drugs can be found, that have indications for UC but are not targeting any of the 30 IBD-associated genes but other genes that are regulated by at least one of the three miRNAs. The drugs are sulfadiazine, azathioprine, methylprednisolone, cortisone acetate, budesonide, prednisone/prednisolone, sulfasalazine, loperamide, and hydrocortisone. These drugs target seven genes, namely *NR3C1*, *CALM1*, *RAC1*, *SLC7A11*, *HTR2A*, *SCN3A*, and *GRIN2A*.

Comorbidity between UC, or any other IBD disorder, and diseases associated with those seven genes would give an indication for a shared underlying mechanism that can be dysregulated by the investigated microRNAs. Comorbidities could not be found, even though evidence for their participation in UC, Crohn's disease, or general IBD exists (*NR3C1* [58], *RAC1* [59], *SLC7A11* [60], *HTR2A* [61]). Some indications are given by carvedilol, a hypertensive drug. It has (pre-)clinical implications for IBD and targets both *HTR2A* and *SLC7A11* [62,63].

This leaves room for further investigation of the mechanistic role of identified genes, especially *SLC7A11* and *HTR2A*, in IBD.

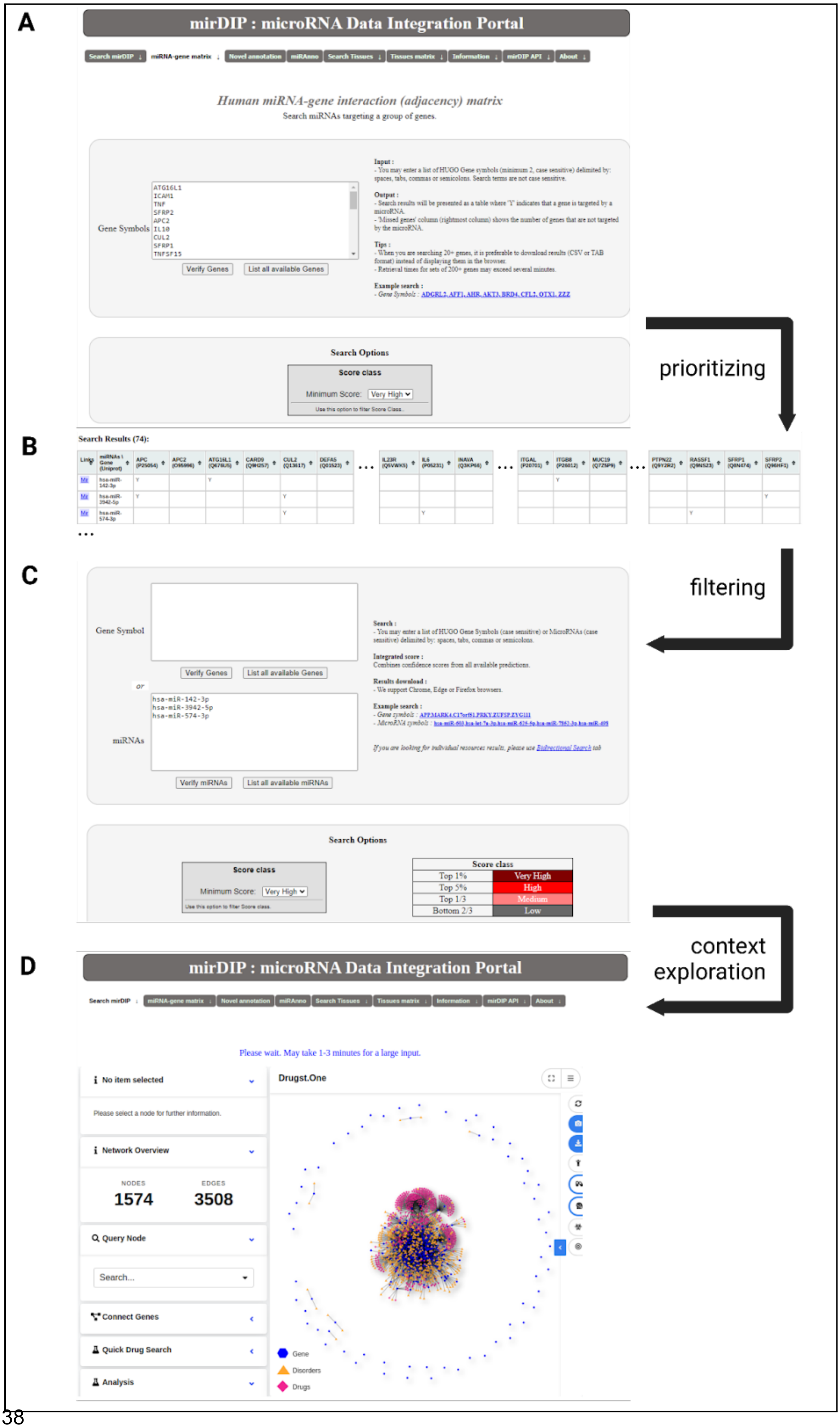
A
mirDIP : microRNA Data Integration Portal
Human miRNA-gene interaction (adjacency) matrix
Search miRNAs targeting a group of genes.
Gene Symbols
Verify Genes
List all available Genes
Search Options
Score class
Minimum Score:
prioritizing
B
Search Results (74):
C
filtering
Gene Symbol
miRNAs
Verify miRNAs
List all available miRNAs
Search Options
Score class
Top 1%
Very High
Top 5%
High
Top 1/3
Medium
Bottom 2/3
Low
D
context exploration
mirDIP : microRNA Data Integration Portal
Please wait. May take 1-3 minutes for a large input.
No item selected
Please select a node for further information.
Network Overview
NODES
1574
EDGES
3508
Query Node
Search...
Connect Genes
Quick Drug Search
Analysis
Drugst.One
Gene
Disorders
Drugs

Figure S3: Workflow using mirDIP portal with Drugst.One plugin to identify microRNA targets for the 30 IBD genes (Table S3), and related drug targets. mirDIP was queried to identify regulating miRNAs, using all databases (**A**). 74 microRNAs were identified, of which only three regulate three IBD-associated genes each (**B**). Using the three microRNAs as a query to mirDIP (**C**) identifies 534 target genes. This set of genes is used to interrogate Drugst.One (**D**).

5. Other Collaborations

The Drugst.One initiative has collaborations with other projects to further extend its capabilities.

Tool	URL	Tool Description	Collaboration
BioCypher [64]	https://biocypher.org	Drugst.One uses BioCypher to facilitate the extensibility of the database and offer additional datasets, e.g. Omnipath	Integration in Drugst.One data warehouse
NDEx IQuery [65]	https://www.ndexbio.org/iquery/	Web tool for pathway and network-based gene set analysis. Allows Drugst.One users to search for curated pathways based on selected genes	Integration in Drugst.One plugin
NDEx [66,67]	https://www.ndexbio.org/	Web platform for storing, sharing, and publishing user-created biological networks.	Integration of “Export to NDEx” function into Drugst.One plugin

Supplementary Table S6. Additional already initiated collaborations that are upcoming improvements to the Drugst.One platform.

References

1. Shows TB, McAlpine PJ. The catalog of human genes and chromosome assignments. A report on human genetic nomenclature and genes that have been mapped in man. Cytogenet Cell Genet. 1978;22: 132–145.

2. The UniProt Consortium. UniProt: the universal protein knowledgebase. Nucleic Acids Res. 2017;45: D158–D169.

3. Cunningham F, Allen JE, Allen J, Alvarez-Jarreta J, Amode MR, Armean IM, et al. Ensembl 2022. Nucleic Acids Res. 2022;50: D988–D995.

4. Maglott D, Ostell J, Pruitt KD, Tatusova T. Entrez Gene: gene-centered information at NCBI. Nucleic Acids Res. 2007;35: D26–31.

5. Mungall CJ, McMurry JA, Köhler S, Balhoff JP, Borromeo C, Brush M, et al. The Monarch Initiative: an integrative data and analytic platform connecting phenotypes to genotypes across species. Nucleic Acids Res. 2017;45: D712–D722.

6. Sadegh S, Skelton J, Anastasi E, Bernett J, Blumenthal DB, Galindez G, et al. Network medicine for disease module identification and drug repurposing with the NeDRex platform. Nat Commun. 2021;12: 6848.

7. Alonso-López D, Campos-Laborie FJ, Gutiérrez MA, Lambourne L, Calderwood MA, Vidal M, et al. APID database: redefining protein–protein interaction experimental evidences and binary interactomes. Database . 2019;2019: baz005.

8. Stark C, Breitkreutz B-J, Reguly T, Boucher L, Breitkreutz A, Tyers M. BioGRID: a general repository for interaction datasets. Nucleic Acids Res. 2006;34: D535–9.

9. Mendez D, Gaulton A, Bento AP, Chambers J, De Veij M, Félix E, et al. ChEMBL: towards direct deposition of bioassay data. Nucleic Acids Res. 2019;47: D930–D940.

10. Davis AP, Grondin CJ, Johnson RJ, Sciaky D, Wiegers J, Wiegers TC, et al. Comparative Toxicogenomics Database (CTD): update 2021. Nucleic Acids Res. 2021;49: D1138–D1143.

11. Freshour SL, Kiwala S, Cotto KC, Coffman AC, McMichael JF, Song JJ, et al. Integration of the Drug–Gene Interaction Database (DGIdb 4.0) with open crowdsource efforts. Nucleic Acids Res. 2020;49: D1144–D1151.

12. Pinero J, Queralt-Rosinach N, Bravo A, Deu-Pons J, Bauer-Mehren A, Baron M, et al. DisGeNET: a discovery platform for the dynamical exploration of human diseases and their genes. Database. 2015. pp. bav028–bav028. doi:10.1093/database/bav028

13. Wishart DS, Feunang YD, Guo AC, Lo EJ, Marcu A, Grant JR, et al. DrugBank 5.0: a major update to the DrugBank database for 2018. Nucleic Acids Res. 2018;46: D1074–D1082.

14. Avram S, Bologa CG, Holmes J, Bocci G, Wilson TB, Nguyen D-T, et al. DrugCentral 2021 supports drug discovery and repositioning. Nucleic Acids Res. 2021;49: D1160–D1169.

15. Carithers LJ, Ardlie K, Barcus M, Branton PA, Britton A, Buia SA, et al. A novel approach to high-quality postmortem tissue procurement: The GTEx project. Biopreserv Biobank. 2015;13: 311–319.

16. Kotlyar M, Pastrello C, Ahmed Z, Chee J, Varyova Z, Jurisica I. IID 2021: towards context-specific protein interaction analyses by increased coverage, enhanced annotation and enrichment analysis. Nucleic Acids Res. 2022;50: D640–D647.

17. Orchard S, Ammari M, Aranda B, Breuza L, Briganti L, Broackes-Carter F, et al. The MIntAct project--IntAct as a common curation platform for 11 molecular interaction databases. Nucleic Acids Res. 2014;42: D358–63.

18. Amberger JS, Bocchini CA, Scott AF, Hamosh A. OMIM.org: leveraging knowledge across phenotype-gene relationships. Nucleic Acids Res. 2019;47: D1038–D1043.

19. Snel B, Lehmann G, Bork P, Huynen MA. STRING: a web-server to retrieve and display the repeatedly occurring neighbourhood of a gene. Nucleic Acids Res. 2000;28: 3442–3444.

20. Junker BH, Koschützki D, Schreiber F. Exploration of biological network centralities with CentiBiN. BMC Bioinformatics. 2006;7: 219.

21. Girvan M, Newman MEJ. Community structure in social and biological networks. Proc Natl Acad Sci U S A. 2002;99: 7821–7826.

22. Rochat Y. Closeness centrality extended to unconnected graphs: The harmonic centrality index. [cited 7 Feb 2023]. Available: https://infoscience.epfl.ch/record/200525/files/[EN]ASNA09.pdf

23. Koschützki D, Schreiber F. Centrality analysis methods for biological networks and their application to gene regulatory networks. Gene Regul Syst Bio. 2008;2: 193–201.

24. Jalili M, Salehzadeh-Yazdi A, Gupta S, Wolkenhauer O, Yaghmaie M, Resendis-Antonio O, et al. Evolution of Centrality Measurements for the Detection of Essential Proteins in Biological Networks. Front Physiol. 2016;7: 375.

25. Zhang H, Fiszman M, Shin D, Miller CM, Rosemblat G, Rindflesch TC. Degree centrality for semantic abstraction summarization of therapeutic studies. J Biomed Inform. 2011;44: 830–838.

26. Tang X, Wang J, Zhong J, Pan Y. Predicting Essential Proteins Based on Weighted Degree Centrality. IEEE/ACM Trans Comput Biol Bioinform. 2014;11: 407–418.

27. List M, Alcaraz N, Dissing-Hansen M, Ditzel HJ, Mollenhauer J, Baumbach J. KeyPathwayMinerWeb: online multi-omics network enrichment. Nucleic Acids Res. 2016;44: W98–W104.

28. Sadegh S, Matschinske J, Blumenthal DB, Galindez G, Kacprowski T, List M, et al. Exploring the SARS-CoV-2 virus-host-drug interactome for drug repurposing. Nat Commun. 2020;11: 3518.

29. Ahmed R, Angelini P, Sahneh FD, Efrat A, Glickenstein D, Gronemann M, et al. Multi-level Steiner Trees. ACM J Exp Algorithmics. 2019;24: 1–22.

30. Gyöngyi Z, Garcia-Molina H, Pedersen J. - Combating Web Spam with TrustRank. In: Nascimento MA, Özsu MT, Kossmann D, Miller RJ, Blakeley JA, Schiefer B, editors. Proceedings 2004 VLDB Conference. St Louis: Morgan Kaufmann; 2004. pp. 576–587.

31. Page L, Brin S, Motwani R, Winograd T. The PageRank Citation Ranking: Bringing Order to the Web. 1999 [cited 7 Feb 2023]. Available: http://ilpubs.stanford.edu:8090/422/

32. Gyongyi Z, Garcia-Molina H, Pedersen J. Error - Stanford InfoLab Publication Server. [cited 30 May 2023]. Available: http://ilpubs.stanford.edu/638/

33. Agrawal M, Kim ES, Colombel J-F. JAK Inhibitors Safety in Ulcerative Colitis: Practical Implications. J Crohns Colitis. 2020;14: S755–S760.

34. Bjerrum JT, Nielsen OH, Riis LB, Pittet V, Mueller C, Rogler G, et al. Transcriptional analysis of left-sided colitis, pancolitis, and ulcerative colitis-associated dysplasia. Inflamm Bowel Dis. 2014;20: 2340–2352.

35. Broom OJ, Widjaya B, Troelsen J, Olsen J, Nielsen OH. Mitogen activated protein kinases: a role in inflammatory bowel disease? Clin Exp Immunol. 2009;158: 272–280.

36. Coskun M, Olsen J, Seidelin JB, Nielsen OH. MAP kinases in inflammatory bowel disease. Clin Chim Acta. 2011;412: 513–520.

37. Sayoc-Becerra A, Krishnan M, Fan S, Jimenez J, Hernandez R, Gibson K, et al. The JAK-Inhibitor Tofacitinib Rescues Human Intestinal Epithelial Cells and Colonoids from Cytokine-Induced Barrier Dysfunction. Inflamm Bowel Dis. 2020;26: 407–422.

38. Armuzzi A, Hart A, Cappelleri JC, Mammar N, Hur P, Hoskin B, et al. Characteristics, clinical outcomes and patient-reported outcomes of patients with ulcerative colitis receiving tofacitinib: a real-world survey in the United States and five European countries. BMC Gastroenterol. 2023;23: 17.

39. Del Sordo R, Lupinacci G, Tanzi G, Bassotti G, Villanacci V. Imatinib and Dasatinib-induced Ulcerative Colitis: Case Report. Inflamm Bowel Dis. 2022;28: e1–e2.

40. Yamauchi K, Inaba T, Colvin HS, Sakakihara I, Yamamoto K, Izumikawa K, et al. Dasatinib-induced colitis: clinical, endoscopic and histological findings. Scand J Gastroenterol. 2022;57: 449–456.

41. Lopez-Millan B, Diaz de la Guardia R, Roca-Ho H, García-Herrero CM, Lavoie JR, Rosu-Myles M, et al. Therapeutic effect of the immunomodulatory drug lenalidomide, but not pomalidomide, in experimental models of rheumatoid arthritis and inflammatory bowel disease. Exp Mol Med. 2017;49: e290.

42. Srinivasan R, Akobeng AK. Thalidomide and thalidomide analogues for induction of remission in Crohn's disease. Cochrane Database Syst Rev. 2009; CD007350.

43. Millrine D, Kishimoto T. A Brighter Side to Thalidomide: Its Potential Use in Immunological Disorders. Trends Mol Med. 2017;23: 348–361.

44. Issac AG, Szafron D, Wei D, McQuade JL, Wang Y. Severe Inflammatory Colitis Related to Encorafenib and Binimetinib following Immune Checkpoint Inhibitor Therapy. Case Rep Gastroenterol. 2022;16: 388–393.

45. Gareb B, Otten AT, Frijlink HW, Dijkstra G, Kosterink JGW. Review: Local Tumor Necrosis Factor-α Inhibition in Inflammatory Bowel Disease. Pharmaceutics. 2020;12. doi:10.3390/pharmaceutics12060539

46. Cui G, Fan Q, Li Z, Goll R, Florholmen J. Evaluation of anti-TNF therapeutic response in patients with inflammatory bowel disease: Current and novel biomarkers. EBioMedicine. 2021;66: 103329.

47. Goenka MK, Kochhar R, Tandia B, Mehta SK. Chloroquine for mild to moderately active ulcerative colitis: comparison with sulfasalazine. Am J Gastroenterol. 1996;91: 917–921.

48. Bak Y-K, Lampe JW, Sung M-K. Effects of dietary supplementation of glucosamine sulfate on intestinal inflammation in a mouse model of experimental colitis. J Gastroenterol Hepatol. 2014;29: 957–963.

49. Shailubhai K, Palejwala V, Arjunan KP, Saykhedkar S, Nefsky B, Foss JA, et al. Plecanatide and dolcanatide, novel guanylate cyclase-C agonists, ameliorate gastrointestinal inflammation in experimental models of murine colitis. World J Gastrointest Pharmacol Ther. 2015;6: 213–222.

50. Izeboud CA, Monshouwer M, van Miert AS, Witkamp RF. The beta-adrenoceptor agonist clenbuterol is a potent inhibitor of the LPS-induced production of TNF-alpha and IL-6 in vitro and in vivo. Inflamm Res. 1999;48: 497–502.

51. Fiebich BL, Collado JA, Stratz C, Valina C, Hochholzer W, Muñoz E, et al. Pseudoephedrine inhibits T-cell activation by targeting NF-κB, NFAT and AP-1 signaling pathways. Immunopharmacol Immunotoxicol. 2012;34: 98–106.

52. Liao J, Keiser JA, Scales WE, Kunkel SL, Kluger MJ. Role of epinephrine in TNF and IL-6 production from isolated perfused rat liver. Am J Physiol. 1995;268: R896–901.

53. Griffin ÉW, Yssel JD, O’Neill E, Ryan KJ, Boyle N, Harper P, et al. The β2-adrenoceptor agonist clenbuterol reduces the neuroinflammatory response, neutrophil infiltration and apoptosis following intra-striatal IL-1β administration to rats. Immunopharmacol Immunotoxicol. 2018;40: 99–106.

54. Wu Z, Kong X, Zhang T, Ye J, Fang Z, Yang X. Pseudoephedrine/ephedrine shows potent anti-inflammatory activity against TNF-α-mediated acute liver failure induced by lipopolysaccharide/D-galactosamine. Eur J Pharmacol. 2014;724: 112–121.

55. Ihara S, Hirata Y, Koike K. TGF-β in inflammatory bowel disease: a key regulator of immune cells, epithelium, and the intestinal microbiota. J Gastroenterol. 2017;52: 777–787.

56. Hauschild A-C, Pastrello C, Ekaputeri GKA, Bethune-Waddell D, Abovsky M, Ahmed Z, et al. MirDIP 5.2: tissue context annotation and novel microRNA curation. Nucleic Acids Res. 2023;51: D217–D225.

57. Yarani R, Shojaeian A, Palasca O, Doncheva NT, Jensen LJ, Gorodkin J, et al. Differentially Expressed miRNAs in Ulcerative Colitis and Crohn’s Disease. Front Immunol. 2022;13: 865777.

58. Skrzypczak-Zielinska M, Gabryel M, Marszalek D, Dobrowolska A, Slomski R. NGS study of glucocorticoid response genes in inflammatory bowel disease patients. Arch Med Sci. 2021;17: 417–433.

59. Seinen ML, van Nieuw Amerongen GP, de Boer NKH, van Bodegraven AA. Rac Attack: Modulation of the Small GTPase Rac in Inflammatory Bowel Disease and Thiopurine Therapy. Mol Diagn Ther. 2016;20: 551–557.

60. Xu S, He Y, Lin L, Chen P, Chen M, Zhang S. The emerging role of ferroptosis in intestinal disease. Cell Death Dis. 2021;12: 289.

61. Alvarado DM, Ciorba MA. Serotonin Receptors Regulate Inflammatory Response in Experimental Colitis. J Nutr. 2020;150: 1678–1679.

62. Ramadan N, Sami S, Elmenshawy S, Salem AE. Possible protective effects of

propranolol and carvedilol and their interaction with mesalazine on experimentally induced ulcerative colitis in male albino rats under supervision of. Unpublished; 2021. doi:10.13140/RG.2.2.22062.38724

63. Murnane KS, Guner OF, Bowen JP, Rambacher KM, Moniri NH, Murphy TJ, et al. The adrenergic receptor antagonist carvedilol interacts with serotonin 2A receptors both in vitro and in vivo. Pharmacol Biochem Behav. 2019;181: 37–45.

64. Lobentanzer S, Aloy P, Baumbach J, Bohar B, Carey VJ, Charoentong P, et al. Democratizing knowledge representation with BioCypher. Nat Biotechnol. 2023. doi:10.1038/s41587-023-01848-y

65. Pillich RT, Chen J, Churas C, Fong D, Ideker T, Liu SN, et al. NDEx IQuery: a multi-method network gene set analysis leveraging the Network Data Exchange. bioRxiv. 2022. p. 2022.10.24.513552. doi:10.1101/2022.10.24.513552

66. Pratt D, Chen J, Pillich R, Rynkov V, Gary A, Demchak B, et al. NDEx 2.0: A Clearinghouse for Research on Cancer Pathways. Cancer Res. 2017;77: e58–e61.

67. Pillich RT, Chen J, Churas C, Liu S, Ono K, Otasek D, et al. NDEx: Accessing Network Models and Streamlining Network Biology Workflows. Curr Protoc. 2021;1: e258.
